# The complete mitochondrial genome of the Variable Platyfish *Xiphophorus variatus*

**DOI:** 10.1080/23802359.2021.1963339

**Published:** 2021-08-13

**Authors:** Anna Nicole Eastis, Kayla Marie Fast, Michael Warren Sandel

**Affiliations:** Department of Biological and Environmental Sciences, University of West Alabama, Livingston, AL, USA

**Keywords:** Oxford Nanopore, *Xiphophorus variatus*, mitochondrial genome, invasive species

## Abstract

We present the complete mitochondrial genome sequence of the Variable Platyfish, *Xiphophorus variatus* (Meek 1904) (Cyprinodontiformes: Poeciliidae). The genome consists of 16,624 bp which encodes 13 protein-coding genes, 22 transfer RNAs, 2 ribosomal RNAs, and 1 control region. Genome-wide nucleotide composition is 27.79% adenine, 31.11% cytosine, 15.63% guanine, and 25.48% thymine. The *X. variatus* mitochondrial genome shares similar GC content and identical gene order and gene strand location with other members of Poeciliidae. The sequence presented herein will be of utility for future phylogenetic and biomedical research and for designing primers for species detection from environmental DNA samples.

The genus *Xiphophorus* contains 26 species of live-bearing platyfish and swordtails and is a member of the order Cyprinodontiformes. *Xiphophorus* species serve as important vertebrate models for biomedical research, sexual selection, and sex determinism (Kallman 1973; Schartl 1995; Kingston et al. 2003; Meyer et al. 2006). The Variable Platyfish, *Xiphophorus variatus* (Meek 1904), is native to watersheds draining to the Gulf of Mexico, from southern Tamaulipas into northern Veracruz states (Page and Burr 1991; Culumber and Rosenthal 2013). The species has been introduced to nonnative watersheds in tropical and subtropical regions of every continent except Antarctica, which is thought to be due to its popularity in the aquarium trade (Cohen et al. 2014). The introduction of *Xiphophorus* species is associated with concurrent declines in the abundance and diversity of native species, which is a cause for concern among conservation organizations aiming to control the spread of invasive species (Máiz-Tomé et al. 2018; Nico 2019). We present a complete mitochondrial genome to aid in noninvasive detection of *X. variatus* in environmental DNA samples and to provide a resource to teams investigating phylogenetics and functional genomics of mitochondrial DNA (mtDNA).

Fin clip samples of *Xiphophorus variatus* strain ‘Zarco’ were obtained from Texas State University Xiphophorus Genetic Stock Center and preserved in 100% ethanol. Specimens were collected from the Arroyo Zarco locality west of Encino, Tamaulipas, Mexico (Walter et al. 2006). Genomic DNA was extracted from a portion of the caudal fin using the Qiagen DNeasy Blood and Tissue kit and was deposited into the Laboratory of AQuatic Evolution (LAQE) at the University of West Alabama (No. 1755Xvariatus, Anna Eastis, eastisa@uwa.edu). DNA quality and quantity were assessed with agarose gel electrophoresis and a NanoDrop spectrophotometer, respectively.

We sequenced the whole mitochondrial genome using a Flongle flow cell utilized for Oxford Nanopore Technologies (ONT, Oxford, UK) MinION, which is a third-generation sequencing technology (Maestri et al. 2019). The reads were assembled to a reference (mtDNA of *Xiphophorus maculatus* – GenBank NC_011379.1) using Geneious software, v11.1.3 (https://www.geneious.com; Kearse et al. 2012). The alignments were checked by eye in Bioedit v7.2.5 (Hall 1999). The genome was annotated using MitoAnnotator through the MitoFish portal (Iwasaki et al. 2013). The mitochondrial genome of *X. variatus* (GenBank accession number MW 934558) has a length of 16,624 bp. The frequencies of adenine, cytosine, guanine, and thymine are 27.79%, 31.11%, 15.63%, 25.48%, respectively, and showed a GC content of 46.73%. Eighteen additional Poeciliidae mitogenomes were used in alignment using the MAFFT online server (Katoh et al. 2019). Maximum-likelihood phylogenetic analysis was conducted in RAxML-HPC 8.2.12 (Stamatakis 2014) and viewed in Dendroscope v3.7.3 (Huson and Scornavacca 2020). The resulting phylogeny, Figure 1, shows the monophyly of *Xiphophorus*, *Poecilia*, *Gambusia*, and *Poeciliopsis*. This is consistent with the results of previous phylogenetic studies of the family Poeciliidae (Pollux et al. 2014; Jeon et al. 2016). Within *Xiphophorus*, most relationships are strongly supported and consistent with previous studies. The position of *X. maculatus*, however, is inconsistent with the mtDNA analysis of Kang et al.(2013) which resolved *X. maculatus* within a clade containing *X. variatus* and all other northern platyfish species. The analysis presented here provides moderate support for a sister relationship between *X. maculatus*, the Southern Platyfish, and *Xiphophorus hellerii*, the Green Swordtail. We expect that increased taxonomic sampling will aid in resolving this node and other relationships within the genus.

**Figure 1.**
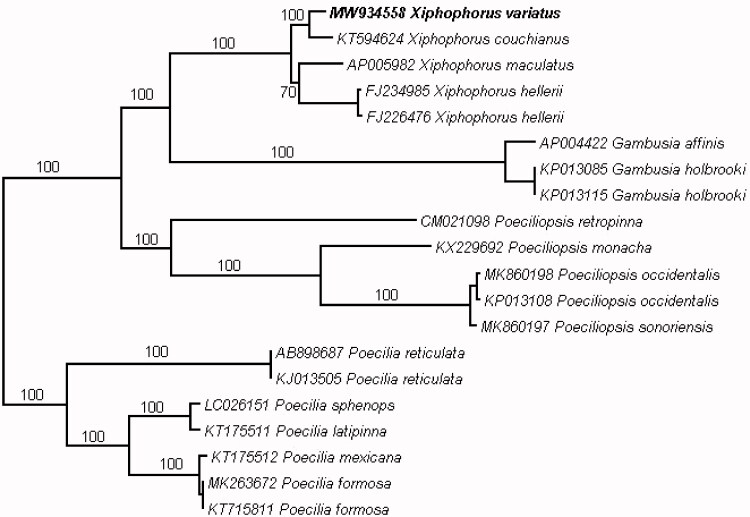
The evolutionary history of Poeciliidae mitogenomes was inferred by using the maximum-likelihood method in the RAxML-HPC BlackBox through the CIPRES portal using 16,944 bp. The resulting file was viewed in Dendroscope. Numbers on branch lengths are bootstrap support values in %. The study species, *Xiphophorus variatus*, is boldened.

## Data Availability

The genome sequence data that support the findings of this study are openly available in GenBank of NCBI at (https://www.ncbi.nlm.nih.gov/) under the accession no. MW934558. The associated BioProject, SRA, and Bio-Sample numbers are PRJNA742674, SRX11313963, and SAMN19969452, respectively.
